# ERK/MAPK Is Essential for Endogenous Neuroprotection in SCN2.2 Cells

**DOI:** 10.1371/journal.pone.0023493

**Published:** 2011-08-17

**Authors:** Sumedha W. Karmarkar, Kathleen M. Bottum, Stacey L. Krager, Shelley A. Tischkau

**Affiliations:** 1 Department of Pharmacology, Southern Illinois University School of Medicine, Springfield, Illinois, United States of America; 2 Department of Internal Medicine, Southern Illinois University School of Medicine, Springfield Illinois, United States of America; Vanderbilt University, United States of America

## Abstract

**Background:**

Glutamate (Glu) is essential to central nervous system function; however excessive Glu release leads to neurodegenerative disease. Strategies to protect neurons are underdeveloped, in part due to a limited understanding of natural neuroprotective mechanisms, such as those present in the suprachiasmatic nucleus (SCN). This study tests the hypothesis that activation of ERK/MAPK provides essential protection to the SCN after exposure to excessive Glu using the SCN2.2 cells as a model.

**Methodology:**

Immortalized SCN2.2 cells (derived from SCN) and GT1-7 cells (neurons from the neighboring hypothalamus) were treated with 10 mM Glu in the presence or absence of the ERK/MAPK inhibitor PD98059. Cell death was assessed by Live/Dead assay, MTS assay and TUNEL. Caspase 3 activity was also measured. Activation of MAPK family members was determined by immunoblot. Bcl2, neuritin and Bid mRNA (by quantitative-PCR) and protein levels (by immunoblot) were also measured.

**Principal Findings:**

As expected Glu treatment increased caspase 3 activity and cell death in the GT1-7 cells, but Glu alone did not induce cell death or affect caspase 3 activity in the SCN2.2 cells. However, pretreatment with PD98059 increased caspase 3 activity and resulted in cell death after Glu treatment in SCN2.2 cells. This effect was dependent on NMDA receptor activation. Glu treatment in the SCN2.2 cells resulted in sustained activation of the anti-apoptotic pERK/MAPK, without affecting the pro-apoptotic p-p38/MAPK. In contrast, Glu exposure in GT1-7 cells caused an increase in p-p38/MAPK and a decrease in pERK/MAPK. Bcl2-protein increased in SCN2.2 cells following Glu treatment, but not in GT1-7 cells; bid mRNA and cleaved-Bid protein increased in GT1-7, but not SCN2.2 cells.

**Conclusions:**

Facilitation of ERK activation and inhibition of caspase activation promotes resistance to Glu excitotoxicity in SCN2.2 cells.

**Significance:**

Further research will explore ERK/MAPK as a key molecule in the prevention of neurodegenerative processes.

## Introduction

Neurodegenerative diseases such as Alzheimer's, Parkinson's, Huntington's and Stroke have no cure, and represent a major source of morbidity and mortality in western society. Once the process of neurodegeneration begins, therapies and treatments to reverse or prevent neuronal loss are scarce. A major factor contributing to the paucity of treatment options is the lack of fundamental understanding of cellular processes leading to cell demise. An additional obstacle is insufficient comprehension of mechanisms utilized by cells to protect themselves from death in the face of the neurotoxic insults [1] that accompany degenerative processes. Excessive glutamate (Glu) release is a primary cause of neuronal death in several neurodegenerative disorders [2], [3], [4]. Thus, the responsiveness of a cell population (such as the SCN2.2 cells) to large amounts of Glu may be key to understanding neuroprotection and neurodegeneration.

The SCN has been widely studied for its role as a circadian pacemaker [5], [6], [7], [8], [9], [10], [11], [12], [13], [14]. Although the SCN is renowned for its resistance to glutamate excitotoxicity [15], [16], [17], [18], [19], [20], mechanisms underlying this endogenous neuroprotection remain obscure. Recently, we demonstrated, for the first time, that the SCN2.2 cell line, which is derived from rat SCN, retains resistance to Glu excitotoxicity, [1]. This study represents an initial foray into determining the mechanisms and signaling pathways involved in SCN2.2 cell resistance to Glu excitotoxicity.

Mitogen-activated protein kinases (MAPKs) are signal transducers that have been implicated in cellular events resulting in both cell death [21] and survival [22]. Of the three major mammalian MAPK proteins, the extracellular regulated kinase/MAPK (ERK/MAPK) pathway is commonly associated with survival [23], whereas p38/MAPK [24] and stress activated protein kinase/Jun N-terminal kinase (SAPK/JNK) pathways are often implicated in cell death [25], [26]. The signal transduction pathways for each of these kinases have been extensively elucidated in cancer studies. Interestingly, however, MAPKs are also essential for regulating physiological responses to light and Glu in the SCN *in vivo* [27]. Therefore, we have explored the roles of MAPKs in SCN2.2 cells in an effort to address whether the mechanistic pathway for endogenous neuroprotection in the SCN2.2 cells depends on the MAPK signaling cascade.

## Results

### ERK/MAPK Inhibitor PD98059 Induces NMDAR-Mediated Cell Death in SCN2.2 Cells

**Figure 1:**
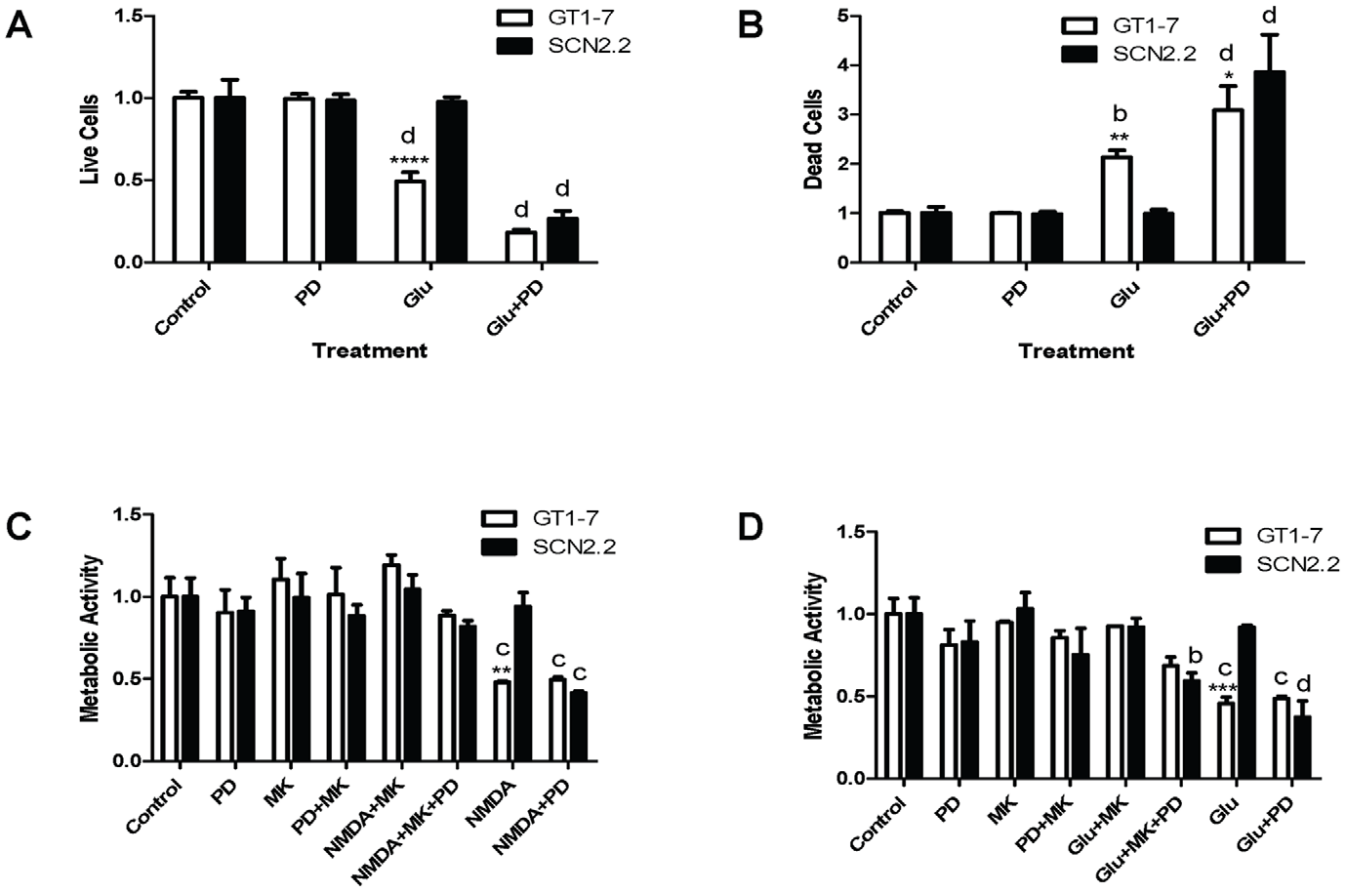
ERK/MAPK Inhibitor PD98059 Induces NMDAR-Mediated Cell Death in SCN2.2 Cells. **A:** Live cells in GT1-7 and SCN2.2 cultures exposed to normal culture conditions (control); 10 µM PD98059 (PD); 10 mM Glu (Glu); or 10 mM Glu+10 µM PD98059 (Glu+PD) for 48 h. PD98059 was added 1 h prior to Glu, and continued throughout Glu treatment. Live cells were measured with the Live/Dead assay. Results are mean ± SD from 2 experiments with n = 18 in each. **B:** Dead cells as measured by Live/Dead assay for same samples as in A. **C:** % Metabolic activity in GT1-7 and SCN2.2 cultures exposed to normal culture conditions (control); 10 µM PD98059 (PD); 10 µM MK-801 (MK); 10 µM PD98059+10 µM MK-801 (PD+MK); 50 µM NMDA+10 µM MK-801 (NMDA+MK); 50 µM NMDA+10 µM MK-801+10 µM PD98059 (NMDA+MK+PD); 50 µM NMDA (NMDA); or 50 µM NMDA+10 µM PD98059 (NMDA+PD) for 48 h. Inhibitors were added 1 h prior to NMDA. Metabolic activity was measured with the MTS assay. Results are mean ± SD from 2 experiments with n = 12 each. **D:** % Metabolic activity in GT1-7 and SCN2.2 cultures exposed to normal culture conditions (control); 10 µM PD98059 (PD); 10 µM MK-801 (MK); 10 µM PD98059+10 µM MK-801 (PD+MK); 10 mM Glu+10 µM MK-801 (NMDA+MK); 10 mM Glu+10 µM MK-801+10 µM PD98059 (Glu+MK+PD); 10 mM Glu (Glu); or 10 mM Glu+10 µM PD98059 (NMDA+PD) for 48 h. Inhibitors were added 1 h prior to Glu. Metabolic activity was measured with the MTS assay. Results are mean ± SD from 2 experiments with n = 12 each. All data analyzed by two-way ANOVA with Bonferroni's post hoc test. Comparisons of GT1-7 vs. SCN2.2 within a treatment group are indicated by: * = p<0.05; ** = p<0.01; *** = p<0.001; **** = p<0.0001. Comparisons of treatment groups vs. control within either GT1-7 or SCN2.2 cells are indicated by: b = p<0.01; c = p<0.001; d = p<0.0001.

For all experiments GT1-7 neurons were used as a positive control as they are susceptible to Glu-induced cell death. GT1-7 and SCN2.2 cells were exposed to the following treatments: 1) normal culture conditions (control); 2) 10 µM of the ERK/MAPK inhibitor PD98059 (PD); 3) 10 mM Glu (Glu) or 4) 10 mM Glu+10 µM PD98059 (Glu+PD). Cell viability was analyzed with the Live/Dead assay. All treatments were for 48 h, except that PD98059 was added 1 h prior to the 48 h Glu incubation in the Glu+PD group. Two-way ANOVA analysis was conducted to assess the effects of cell type and treatment, as well as the interaction of these two factors. Results of overall two-way ANOVA analysis are presented in Table 1. Both cell type (GT1-7 or SCN2.2) and treatment (control, PD, Glu or Glu+PD) as well as the interaction of cell type and treatment had significant effects on the number of live cells. Bonferroni's post hoc test showed that GT1-7 live cells were significantly decreased from control in both the Glu and Glu+PD treatment groups (Figure 1A, d = p<0.0001), whereas SCN2.2 live cells were significantly decreased only in the Glu+PD treatment group when compared to control (Figure 1A, d = p<0.0001). Fold change from control for GT1-7 vs. SCN2.2 live cells were significantly different from each other only for the Glu treatment group (Figure 1A, **** = p<0.0001); with all other treatment conditions there was no significant difference between the cell lines.

**Table 1:** Results of Two-way ANOVA Analysis.

Figure	Cell Type (GT1-7 vs. SCN2.2)	Treatment (condition or time vs. control)	Interaction of Cell Type and Treatment
1A; Live Cells	p<0.0001	p<0.0001	p<0.0001
1B; Dead Cells	ns (p = 0.48)	p<0.0001	p<0.01
1C; MTS assay	ns (p = 0.82)	p<0.0001	p<0.01
1D; MTS assay	ns (p = .28)	p<0.0001	p<0.01
3A; pERK/MAPK	p<0.0001	p<0.0001	p<0.0001
3B; p-p38/MAPK	p<0.0001	ns (p = 0.17)	ns (p = 0.09)
3C; p-SAPK/JNK	p<0.0001	p<0.05	p<0.01
5A; Neuritin	p<0.0001	p<0.05	ns (p = 0.10)
5B; Bcl2	ns (p = 0.32)	p<0.0001	p<0.0001
5C; Bid	p<0.0001	p<0.0001	p<0.0001

Two-way ANOVA analysis with Bonferroni's post hoc correction was used to analyze data in figures 1, 3 and 5. Results of the overall analysis are presented; post hoc analysis p values are given in the text and figures. Statistics were done with GraphPad Prism 5.0.

Similar two-way ANOVA analysis of dead cell results demonstrated a significant effect of treatment (control, PD, Glu or GLU+PD) but not cell type (GT1-7 or SCN2.2); the interaction of cell type and treatment also significantly affected the number of dead cells (Table 1). Bonferroni's post hoc test determined that among the GT1-7 neurons, both the Glu treatment (Figure 1B, b = p<0.01) and the Glu+PD treatment (Figure 1B, d = p<0.0001) resulted in an increased number of dead cells compared to control. Within the SCN2.2 cells however, only the control vs. Glu+PD treatment produced increased dead cells (Figure 1B, d = p<0.0001). Bonferroni's post hoc comparison for fold change showed fewer dead cells in the SCN2.2 vs. GT1-7 cells in the Glu treatment group (Figure 1B, ** = p<0.01); but following the Glu+PD treatment, SCN2.2 cells displayed higher numbers of dead cells than the GT1-7 cells (Figure 1B, * = p<0.05).

The results from the Live/Dead assay are consistent with previous findings from our lab [1], demonstrating that 48 h of 10 mM Glu treatment alone induced cell death in GT1-7 but not in the SCN2.2 cells (Figure 1A and 1B). However, 1 h pre-treatment with 10 µM of the ERK/MAPK inhibitor PD98059 and 48 h concurrent treatment with PD98059 and Glu resulted in a significant decrease in live SCN2.2 cells (Figure 1A) as well as a concomitant increase in dead cells (Figure 1B). Thus, inhibiting ERK/MAPK activity compromised the endogenous neuroprotective mechanism in SCN2.2 against Glu toxicity.

To determine whether NMDAR-mediated signaling was involved in the increased susceptibility to excitotoxicity in SCN2.2 cells exposed to the ERK/MAPK inhibitor PD98059, cells were pre-treated for 1 h with an NMDAR blocker, MK-801, before receiving the excitotoxin NMDA plus PD98059 and MK-801 for 48 h, with appropriate controls. GT1-7 cells were used as a positive control. The experiment included eight treatment groups for each cell type, as follows: 1) Untreated control (control); 2) 10 µM PD98059 (PD); 3) 10 µM MK-801 (MK); 4) 10 µM PD98059+10 µM MK-801 (PD+MK); 5) 50 µM NMDA+10 µM MK-801 (NMDA+MK); 6) 50 µM NMDA+10 µM MK-801+10 µM PD98059 (NMDA+MK+PD); 7) 50 µM NMDA (NMDA) and 8) 50 µM NMDA+10 µM PD98059 (NMDA+PD). Cell viability was determined using the MTS assay to measure cellular metabolic activity. The results were analyzed with two-way ANOVA, using Bonferroni's post hoc test. There was a significant effect of treatment (control, PD, MK, PD+MK, NMDA+MK, NMDA+MK+PD, NMDA or NMDA+PD), but not cell type (GT1-7 or SCN2.2) on metabolic activity in these cells (Table 1). The interaction between treatment and cell type on metabolic activity was also significant (Table 1). Within the GT1-7 cells, both the NMDA and the NMDA+PD treatment groups had significantly reduced metabolic activity compared to controls (Figure 1C, c = p<0.001); whereas among the SCN2.2 cells only the NMDA+PD treatment group showed reduced metabolic activity compared to control (Figure 1C, c = p<0.001). Additional Bonferroni's post hoc analysis compared fold change for GT1-7 vs. SCN2.2 cells in each treatment group. The GT1-7 cells had reduced metabolic activity compared to SCN2.2 cells only for the NMDA treatment group (Figure 1C, ** = p<0.01). As expected, NMDA treatment for 48 h reduced metabolic activity in the GT1-7 cells as compared with control, but this reduction was prevented by MK-801. Also as expected, SCN2.2 cell metabolic activity remained unaffected by NMDA treatment. However, PD98059 severely reduced metabolic activity of NMDA-treated SCN2.2 cells. The effect of NMDA and PD98059 on SCN2.2 cells was prevented by MK-801. Thus, NMDARs may lie upstream of ERK/MAPK activation in SCN2.2 cell resistance to excitotoxicity.

We performed similar experiments in which NMDA was replaced by glutamate (Figure 1D). The results were analyzed with two-way ANOVA, using Bonferroni's post hoc test. There was a significant effect of treatment (control, PD, MK, PD+MK, Glu+MK, Glu+MK+PD, Glu or Glu+PD), but not cell type (GT1-7 or SCN2.2) on metabolic activity in these cells (Table 1). The interaction between treatment and cell type on metabolic activity was significant (Table 1). Within the GT1-7 cells, both the Glu and the Glu+PD treatment groups had significantly reduced metabolic activity compared to controls (Figure 1D, c = p<0.001); whereas in the SCN2.2 cells the Glu group was unchanged from control, but both the Glu+MK+PD and the Glu+PD treatment groups showed reduced metabolic activity compared to control (Figure 1D, b = p<0.01 and d = p<0.0001). Thus in the SCN2.2 cells, MK-801 only partially abolished the effects of Glu+PD. This suggests that SCN2.2 cell death in the Glu+PD treatment group could be mediated in part by a non-NMDA pathway.

### ERK/MAPK inhibition induces cell death in SCN2.2 cells via an apoptotic pathway

To explore whether cell death caused by excitotoxicity in the presence of the ERK/MAPK blocker PD98059 occurs via apoptosis, the following treatments were applied to cultured cells: 1) untreated control (control); 2) 10 µM PD98059 (PD); 3) 10 mM Glu and 4) 10 mM Glu+10 µM PD98059 (Glu+PD). PD98059 was added 1 h prior to Glu and continued throughout the 48 h treatment period. Apoptosis was assessed in 3 ways: 1) cleaved caspase 3 immunocytochemistry; 2) TUNEL and 3) caspase 3 activity assay. All 3 experimental methods produced consistent results.

**Figure 2:**
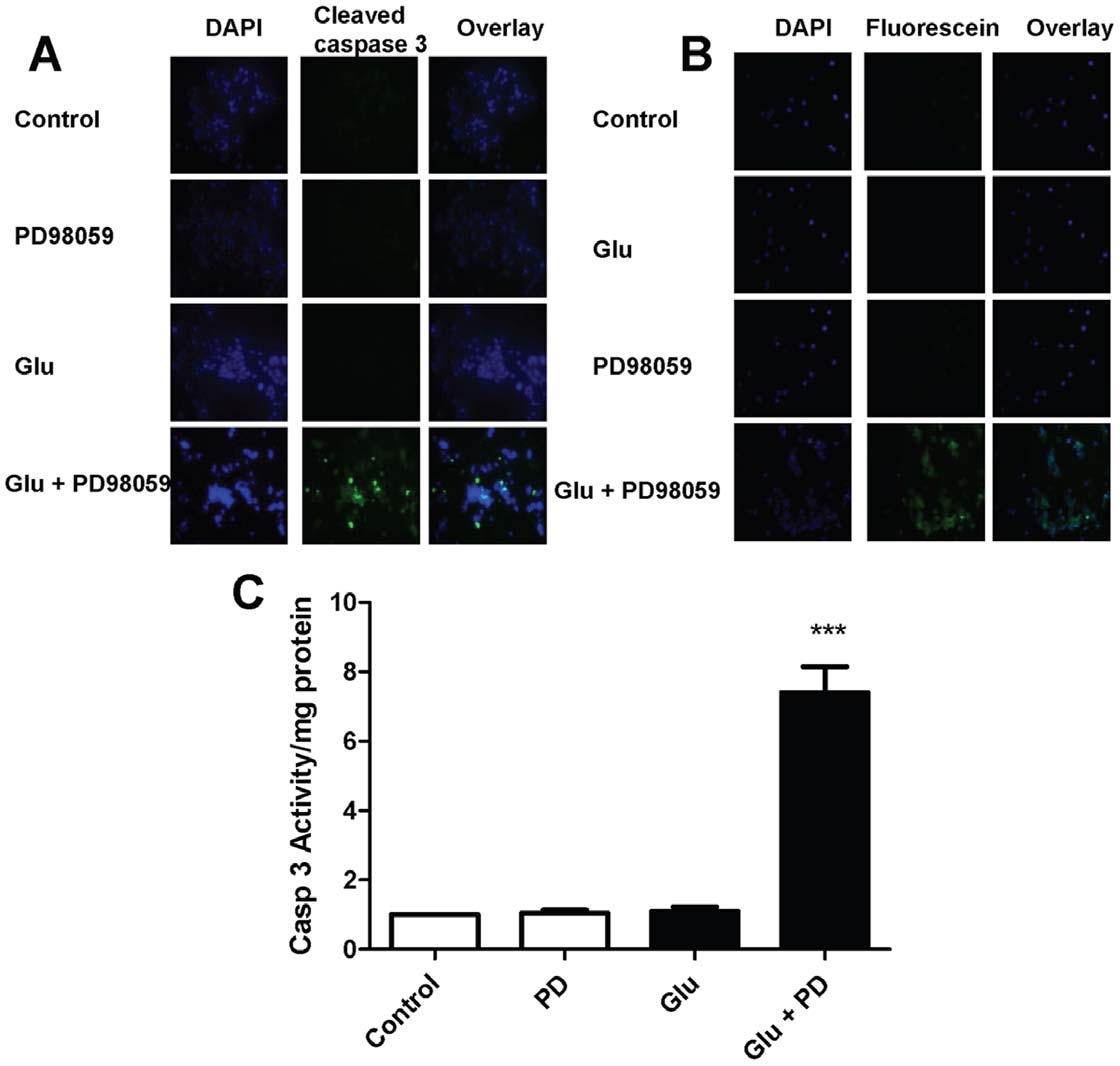
ERK/MAPK Inhibition Induces Cell Death in SCN2.2 Cells via an Apoptotic Pathway. **A:** Immunocytochemistry for activated caspase 3 in SCN2.2 cells exposed to: untreated control (control); 10 µM PD98059 (PD); 10 mM Glu (Glu) or 10 mM Glu+10 µM PD98059 (Glu+PD) for 48 h. Results from typical experiments are shown; 3 different experiments with n = 2 for each experiment were performed. **B:** TUNEL assay in SCN2.2 cells treated with the same conditions as in A. Results from typical experiments are shown; 3 different experiments with n = 2 for each experiment were performed. **C:** Caspase 3 activity in SCN2.2 cells treated with the same conditions as in A. Results are mean ± SEM for 3 experiments with n = 2 each. Comparisons of treatment groups vs. control in C were analyzed with repeated measures ANOVA and Tukey's post hoc comparison. *** = p<0.001.

Treatment of SCN2.2 cells with PD98059+Glu resulted in increased cleaved caspase 3 immunostaining (Figure 2A) and increased TUNEL-positive cells (Figure 2B). Similarly, caspase 3 activity increased significantly in SCN2.2 cells when Glu was added in the presence of PD98059 (Figure 2C, *** = p<0.001) as measured by repeated measures ANOVA with Tukey's post hoc comparison. Control medium replacement, PD98059 alone and Glu alone produced no significant change in any of these parameters in SCN2.2 cells.

### Preferential activation of ERK/MAPK in response to Glu in SCN2.2 cells

**Figure 3:**
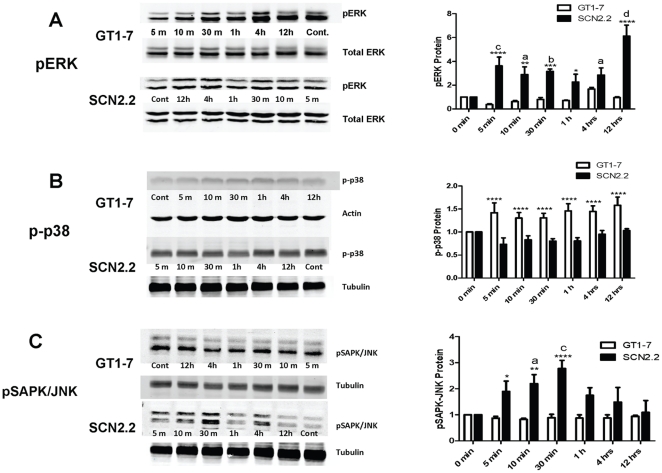
Preferential Activation of ERK/MAPK in Response to Glu in SCN2.2 Cells. **A:** Immunoblot for pERK/MAPK in GT1-7 and SCN2.2 cells exposed to 10 mM Glu for 0 min (control), 5 min, 10 min, 30 min, 1 h, 4 h and 12 h. Sample blots are shown on the left, quantification of blots are displayed on the right. **B:** Immunoblot for p-p38/MAPK in GT1-7 and SCN2.2 cells exposed to 10 mM Glu for the same time periods as in A. Sample blots are shown on the left, quantification of blots are displayed on the right. **C:** Immunoblot for pSAPK-JNK/MAPK in GT1-7 and SCN2.2 cells exposed to 10 mM Glu for the same time periods as in A. Sample blots are shown on the left, quantification of blots are displayed on the right. All experiments were repeated 3 or 4 times, with n = 1 per experiment. Data were analyzed by two-way ANOVA with post hoc Bonferroni's test. For pERK/MAPK and pSAPK-JNK/MAPK where two-way ANOVA showed a significant interaction between cell type and treatment time, comparisons of GT1-7 vs. SCN2.2 within a time point are indicated by: * = p<0.05; ** = p<0.01; *** = p<0.001, **** = p<0.0001. For pERK/MAPK and pSAPK-JNK/MAPK comparisons of time points vs. control (0 min) within either GT1-7 or SCN2.2 cells are indicated by: a = p<0.05; b = p<0.01; c = p<0.001; d = p<0.0001. For p-p38/MAPK, where two-way ANOVA did not show a significant interaction between cell type and treatment time, comparison between cell types as a whole (not broken down by treatment time) is indicated by **** = p<0.001.

**Figure 4:**
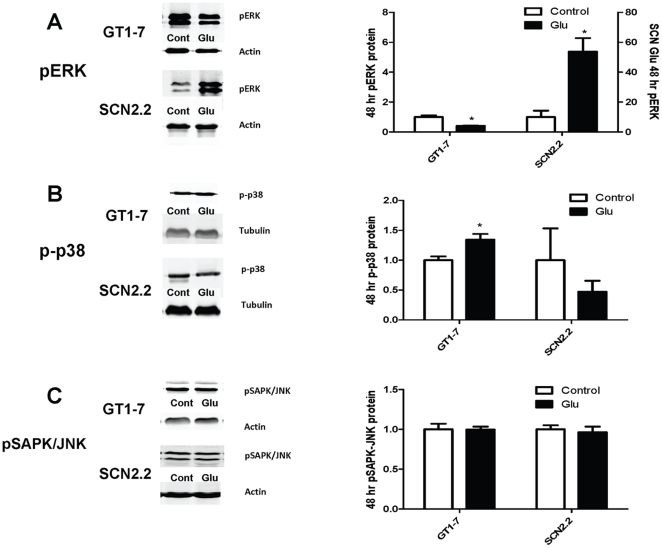
Activation of pERK/MAPK is Sustained for 48 h in SCN2.2 Cells Exposed to Glu. **A:** Immunoblot for pERK/MAPK in GT1-7 and SCN2.2 cells treated with media change (control) or 10 mM Glu for 48 h. Sample blots are shown on the left, quantification of blots are displayed on the right. **B:** Immunoblot for p-p38/MAPK in GT1-7 and SCN2.2 cells treated with media change (control) or 10 mM Glu for 48 h. Sample blots are shown on the left, quantification of blots are displayed on the right. **C:** Immunoblot for pSAPK-JNK/MAPK in GT1-7 and SCN2.2 cells treated with media change (control) or 10 mM Glu for 48 h. Sample blots are shown on the left, quantification of blots are displayed on the right. All experiments were repeated 3 or 4 times, with n = 1 per experiment. Data were analyzed by paired samples *t* test for control vs. 48 h; * = p<0.05.

Immunoblot analysis allowed comparisons of pERK/MAPK, p-p38/MAPK and pSAPK-JNK/MAPK in response to Glu in SCN2.2 and GT1-7 cells. SCN2.2 and GT1-7 cells were treated with 10 mM Glu for different lengths of time (control = 0 min, 5 min, 10 min, 30 min, 1 h, 4 h and 12 h), and cells were collected for immunoblot analysis of pERK/MAPK, p-p38/MAPK and p-SAPK/JNK/MAPK. Results were analyzed by two-way ANOVA with Bonferroni's post hoc test with treatment and cell type as the dependent variables. pERK/MAPK was significantly affected by both cell type (GT1-7 or SCN2.2) and treatment time (control, 5 min, 10 min, 30 min, 1 h, 4 h or 12 h); the interaction between these two factors was also significant (Table 1). ERK/MAPK phosphorylation did not significantly change in GT1-7 cells compared to control for up to 12 h (Figure 3A). After 48 h, a significant decrease in pERK/MAPK was observed in GT1-7 cells compared to control by *t*-test (Figure 4A, * = p<0.05). In contrast, Glu treatment strongly and consistently induced phosphorylation of ERK/MAPK in SCN2.2 at 5 min (Figure 3A, c = p<0.001), 10 min (Figure 3A, a = p<0.0.05), 30 min (Figure 3A, b = p<0.01), 4 h (Figure 3A, a = p<0.05), and 12 h (Figure 3A, d = p<0.0001) compared to control. Increased SCN2.2 pERK/MAPK was maintained at 48 h when control and 48 h samples were compared by *t*-test (Figure 4A, * = p<0.05). A significantly higher induction of pERK/MAPK was observed in SCN2.2 vs. GT1-7 cells at multiple time points including: 5 min (Figure 3A, **** = p<0.0001); 10 min (Figure 3A, ** = p<0.01); 30 min (Figure 3A, *** = p<0.0001); 1 h (Figure 3A, * = p<0.05) and 12 h (Figure 3A, **** = p<0.0001).

Simultaneously, we also probed for p-p38/MAPK and analyzed results by two-way ANOVA with treatment and cell type as the dependent variables. p-p38/MAPK was significantly affected by cell type (GT1-7 or SCN2.2) but not treatment time (control, 5 min, 10 min, 30 min, 1 h, 4 h or 12 h); the interaction between these two factors was not significant (Table 1). Because the interaction of the two factors was not significant, it can only be said that the average of all GT1-7 p-p38/MAPK was greater than the average of all SCN2.2 p-p38/MAPK (p<0.001). There was no significant difference between any time points for either cell type. Comparison of the GT1-7 48 h Glu treatment to control by *t*-test indicated that p-p38/MAPK was significantly higher after treatment (Figure 4B, p<0.05). In contrast, Glu had no significant effect on p-p38/MAPK compared to control in SCN2.2 cells (Figure 3B and Figure 4B).

We also probed for pSAPK/JNK and analyzed results by two-way ANOVA with Bonferroni's post hoc test using treatment and cell type as the dependent variables. pSAPK/JNK was significantly affected by cell type (GT1-7 or SCN2.2) and treatment time (control, 5 min, 10 min, 30 min, 1 h, 4 h or 12 h); the interaction between these two factors was also significant (Table 1). Glu treatment did not significantly alter pSAPK/JNK/MAPK in GT1-7 cells (Figure 3C and Figure 4C). In contrast, Glu treatment caused a transient increase in pSAPK/JNK/MAPK in SCN2.2 cells only at 10 min (Figure 3 C, a = p<0.05) and 30 min (Figure 3C, c = p<0.01) compared to control (Figure 3C and Figure 4C).

### Cell signaling downstream of ERK/MAPK pathway activation in cell survival promotion

**Figure 5:**
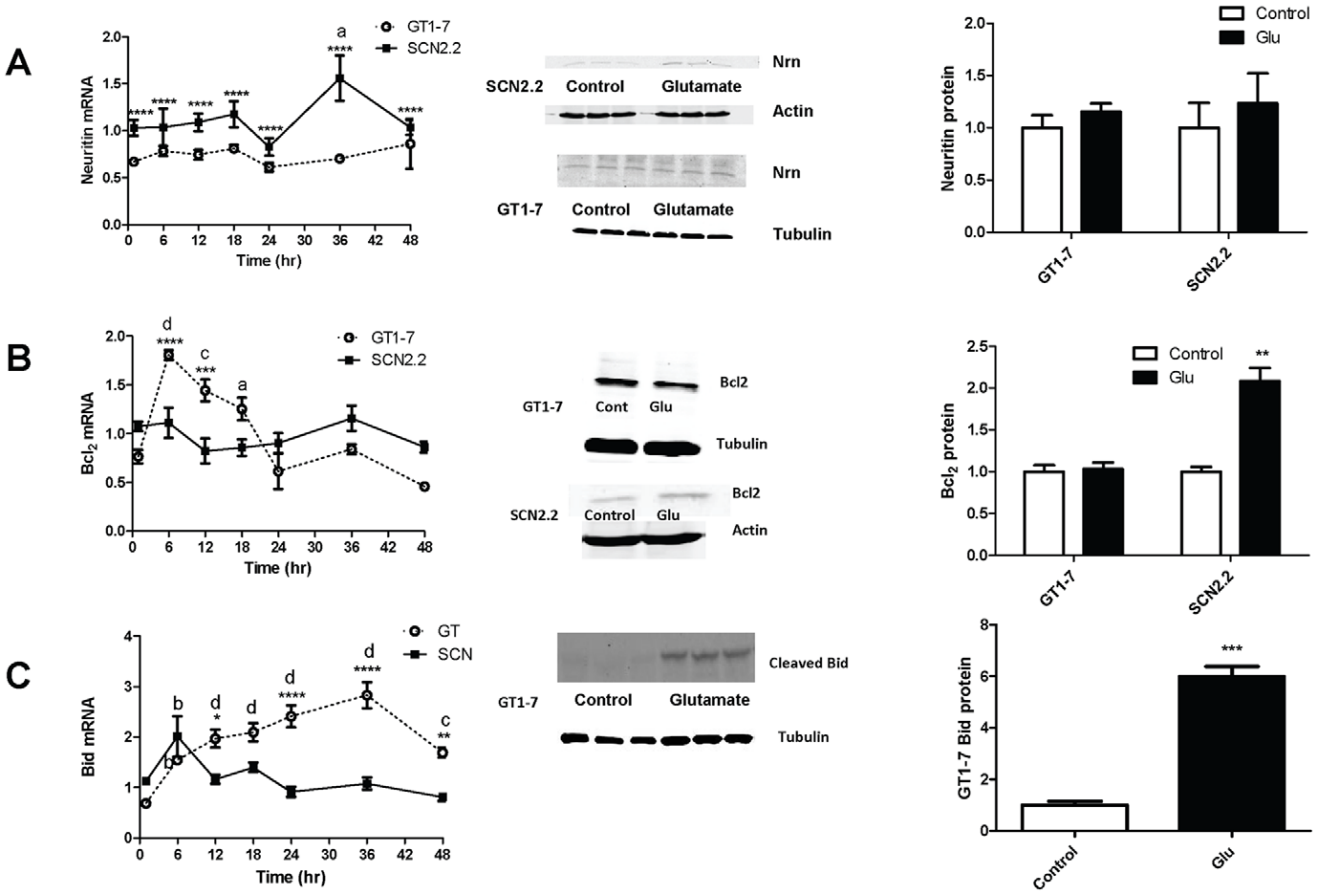
Cell Signaling Downstream of ERK/MAPK Pathway. **A:** mRNA for neuritin in GT1-7 and SCN2.2 cells treated with media change (control) or 10 mM Glu for 6, 12, 18, 24, 36 and 48 h. Immunoblots for neuritin protein in GT1-7 and SCN2.2 cells treated with Glu or control for 48 h are also shown, along with their quantification. **B:** mRNA for Bcl2 in GT1-7 and SCN2.2 cells treated in the same way as in A. Immunoblots for Bcl2 protein in GT1-7 and SCN2.2 cells treated with Glu or control for 48 h are also shown, along with their quantification. **C:** mRNA for Bid in GT1-7 and SCN2.2 cells treated in the same was as in A. Immunoblots for cleaved Bid protein in GT1-7 cells treated with Glu or control for 48 h are also shown, along with their quantification. No suitable antibody was found for cleaved Bid in SCN2.2. PCR data is the average of 2 experiments with n = 3 for each experiment. The Immunoblots are the average of 3 to 4 experiments with n = 1 each. mRNA data were analyzed by two-way ANOVA with Bonferroni's post hoc comparison; immunoblot data were compared using paired *t* test. For Bcl2 and Bid, where two-way ANOVA was significant for an interaction between cell type and treatment time, comparisons of GT1-7 mRNA vs. SCN2.2 mRNA within a time point are indicated by: * = p<0.05; ** = p<0.01; *** = p<0.001, **** = p<0.0001. Comparisons of time points vs. control (0 min) within either GT1-7 or SCN2.2 cells are indicated by: a = p<0.05; b = p<0.01; c = p<0.001; d = p<0.0001. For Neuritin, where two-way ANOVA did not show a significant interaction between cell type and treatment time, comparison between cell types as a whole (not broken down by treatment time) is indicated by **** = p<0.001, and comparison between time points as a whole (not broken down by cell type) is indicated by a = p<0.05. Protein data were analyzed by paired samples t test for control vs. 48 h; ** = p<0.01; *** = p<0.001.

To determine what signaling molecules activated downstream of ERK/MAPK might be associated with protection in SCN2.2 cells, changes in transcript levels for several prosurvival and proapoptotic factors were examined by qPCR and changes in protein levels were assessed with immunoblot following incubation with 10 mM Glu vs. media change (control). Neuritin plays a role in neurite outgrowth and synapse formation. Neuritin mRNA results were analyzed by two-way ANOVA using treatment and cell type as the dependent variables. Neuritin mRNA was significantly affected both by cell type (GT1-7 or SCN2.2) and treatment time (control, 6, 12, 18, 24, 36 or 48 h); however the interaction between these two factors was not significant (Table 1). Thus it can only be said that neuritin mRNA in SCN2.2 (irrespective of treatment time) was significantly higher than Neuritin mRNA in all GT1-7 (p<0.0001) (Figure 5A). Neuritin mRNA (ignoring cell type) was significantly increased at 36 h compared to control (Figure 5A, a = p<0.05). Neuritin protein was measured after 48 h incubation with Glu and compared with control by *t*-test. Neither GT1-7 nor SCN2.2 cells showed a significant change in neuritin protein following 48 hr incubation with 10 mM Glu.

Bcl2 is a well known anti-apoptotic factor associated with cell survival; it blocks the activation of caspases and prevents apoptosis. Bcl2 mRNA was analyzed by two-way ANOVA with Bonferroni's post hoc test using treatment and cell type as the dependent variables. Treatment time (control vs. 6, 12, 18, 24, 36 and 48 h), but not cell type (GT1-7 vs. SCN2.2), significantly influenced Bcl2 mRNA levels; the interaction between treatment time and cell type was also significant (Table 1). Bcl2 mRNA initially increased in GT1-7 time points compared with control at 6 h (Figure 5B, d = p<0.0001), 12 h (Figure 5B, c = p<0.01) and 18 h (Figure 5B, a = p<0.05). At all other time points Bcl2 mRNA was not increased vs. control (Figure 5B). There was no significant change in SCN2.2 Bcl2 mRNA at any time point (Figure 5B). After both 6 h (Figure 5B, **** = p<0.0001) and 12 h (Figure 5, *** = p<0.001) incubation with Glu, GT1-7 Bcl2 mRNA was significantly increased vs. SCN2.2 Bcl2. At all other time points there was no difference between GT1-7 and SCN2.2 Bcl2 mRNA (Figure 5B). Bcl2 protein was measured after 48 h incubation with Glu and compared with control by *t*-test and levels did not change in GT1-7 cells (Figure 5B). Surprisingly, although SCN2.2 mRNA for Bcl2 did not change over a 48 h period, Bcl2 protein levels did significantly increase in the 48 h treatment vs. control (Figure 5B, ** = p<0.01).

Bid is a pro-apoptotic molecule; cleavage of Bid heralds apoptotic processes that are dependent on the mitochondrial pathway. Bid mRNA was analyzed by two-way ANOVA with Bonferroni's post hoc test using treatment and cell type as dependent variables. Both treatment time (control vs. 6, 12, 18, 24, 36 and 48 h) and cell type (GT1-7 vs. SCN2.2) significantly influenced Bid mRNA levels; the interaction between treatment time and cell type was also significant (Table 1). Bid mRNA showed a sustained increased in GT1-7 cells at 6 h (Figure 5C, b = p<0.01), 12 h (Figure 5C, d = p<0.0001), 18 h (Figure 5C, d = p<0.0001), 24 h (Figure 5C, d = p<0.0001), 36 h (Figure 5C, d = p<0.0001) and 48 h compared to the control (Figure 5C, c = p<0.001). Thus at all times tested Bid mRNA was higher in GT1-7 treated cells vs. control. At several time points Bid mRNA was higher in GT1-7 cells compared to SCN2.2, including 12 h (Figure 5C, * = p<0.05), 24 h (Figure 5C, **** = p<0.0001), 36 h (Figure 5C, **** = p<0.0001) and 48 h (Figure 5C, ** = p<0.01). GT1-7 cleaved Bid protein was measured after 48 h incubation with Glu and compared with control by *t*-test; levels of cleaved Bid protein were significantly higher after 48 h Glu treatment vs. control (Figure 5C, *** = p<0.001). Unfortunately, we were unable to obtain a satisfactory antibody against cleaved bid in rats to test cleaved Bid protein levels in SCN2.2 cells.

## Discussion

The SCN is the master biological clock of the body [5], [6], [28]. As such, the SCN must communicate with the external environment to assure that internal processes remain aligned with the environmental light∶dark cycle. Activation of the retinohypothalamic tract by light releases Glu onto the SCN [29], leading to activation of NMDARs on the postsynaptic SCN neurons [30], [31] to produce a calcium influx into the cells. Within the SCN neurons, nocturnal light activates ERK/MAPK [32], [33]; furthermore, blocking ERK/MAPK activation prevents the light response within the SCN neurons [27]. Thus, both glutamate and ERK/MAPK are important signal transducers required for gating mechanisms involved in clock function.

Induction of ERK/MAPK is commonly associated with cell survival [34], [35], [36] through activation of 2 different signaling molecules: 1) cAMP response element binding protein (CREB) (cAMP = cyclic adenosine monophosphate) [33]; and 2) mammalian target of rapamycin (mTOR) [33], [37]. CREB activation is important in transcription of genes, many of which promote cell survival; mTOR similarly supports survival via translational control [38]. Correspondingly, in the SCN, inhibition of CREB or mTOR blocks the physiological effects of nocturnal light [37]. Previously, we demonstrated that the SCN neurons are resistant to Glu excitotoxicity [1]. Because ERK/MAPK is fundamental to transducing the glutamatergic signal in the SCN, we hypothesized that ERK/MAPK activation may also serve to allow SCN2.2 cells to survive the Glu barrage that would be devastating to many other neuronal types.

Our results indicate that resistance to Glu excitotoxicity in SCN2.2 cells is indeed dependent on ERK/MAPK signaling. SCN2.2 cell viability is unaffected by 10 mM Glu (Figure 1). However, pre-treatment of SCN2.2 cells with an ERK/MAPK inhibitor (PD98059) renders these cells susceptible to the excitotoxic effects of Glu (Figure 1), and leads to activation of caspase-dependent apoptosis (Figures 2 and 3). To the best of our knowledge, this is the first report of a signal transducer that plays a role conferring resistance against Glu insult in SCN2.2 cells.

Although extensively studied for promoting cell survival and inhibiting apoptosis in cancer cells [39], more recent studies demonstrate the neuroprotective effects of ERK/MAPK activity in neuronal systems. ERK/MAPK activation reduces neuronal damage incurred by ischemia-hypoxia. BDNF treatment results in ERK/MAPK activation up to 12 h following ischemic-hypoxic injury in neonatal brain and reduces the lesioned area as compared to vehicle treatment alone; this is prevented by inhibition of ERK/MAPK [40]. BDNF treatment prevents camptothecin-induced cortical neuronal death, in an ERK/MAPK-dependent manner [41]. Furthermore, resveratrol and fisetin prevent neuronal death in a Huntington's disease model via ERK/MAPK activation [42]. Reperfusion injury following spinal-cord ischemia can be reduced by a post-conditioning mechanism that requires ERK/MAPK [43]. In axotomized retinal ganglion neurons neurodegeneration can be prevented by VEGF (vascular endothelial growth factor) via ERK/MAPK activation [44]. Finally, estrogen-dependent activation of ERK/MAPK inhibits cell death in hippocampal neurons after global cerebral ischemia [45].

Most of the studies highlighted above reported a single time-point of ERK/MAPK activation. In the current study, we have established a time-course for ERK/MAPK activation in SCN2.2 and GT1-7 cells. ERK/MAPK activation in response to Glu is initially inhibited in the GT1-7 neurons and though it is increased at 4 h, it falls to control levels at the end of 12 h (Fig. 3A) and remains low at 48 h (Fig. 4A). However, SCN2.2 cells display a sustained increase in ERK/MAPK up to 48 h (Fig. 3A and 4A), which might be interpreted as a continuous presence of neuroprotective factor (ERK/MAPK) in SCN2.2 cells in response to Glu. It should be noted here that ERK/MAPK activation can also be deleterious to neurons. Some studies show that inhibition of ERK/MAPK activation prevents neuronal death (reviewed in Zhuang S; Perspectives in Pharmacology; 2006). In our hands, basal pERK levels were also much lower in the SCN2.2 untreated cells as compared to the GT1-7 basal levels. The reasons for this remain unclear.

Because the two other MAPK pathways have also been coupled to cell death or proliferation, we examined Glu-induced activation of the p38/MAPK and SAPK/JNK. Although consensus dictates that p38/MAPK mediates cell death [24], [46], [47], contradictory studies suggest a cell survival role for p38 [48]. We found that phosphorylated (activated) p38 levels did not change with Glu treatment in the SCN2.2 cells in the short time-course (Fig. 3B) or at 48 h (Fig. 4B). However, a significant increase in p-p38 levels in the GT1-7 cells during the short time-course (Fig. 3B) and at 48 h (Fig. 4B) was observed. These data are highly suggestive of preferential activation of a pro-survival ERK/MAPK pathway in SCN2.2 cells as opposed to pro-apoptotic p38/MAPK activation in the GT1-7 cells.

Diverse studies paradoxically implicate the SAPK/JNK pathway in both apoptosis [26], [49] and cell survival [50]. Neurodegeneration in Alzheimer's disease has been related to long-term activation of SAPK/JNK [51]. Overactivation of JNK is associated with glial enlargement [52] and mediation of beta-amyloid toxicity (Hashimoto, 2003). These responses seem to be triggered by long-term activation of SAPK/JNK [53], [54]. However, other studies indicate that a relatively short-term activation of SAPK/JNK is essential for neurite outgrowth [55], [56] [57], neuronal development, and neuronal regeneration [57].

In the current study, phosphorylated (activated) SAPK/JNK levels did not change with Glu treatment in the GT1-7 cells (Fig. 3C and 4C); however, pSAPK/JNK was transiently increased in the SCN2.2 cells peaking at 30 min and then returning to baseline levels (Fig. 3C) and remaining low up to 48 h (Fig. 4C). We speculate that it is the ability of the SCN2.2 cells to respond to stress through transient pSAPK/JNK activation which may, in turn, activate more long-term mechanisms that prevent these cells from undergoing apoptosis in the face of glutamatergic challenge.

Signaling downstream of the ERK/MAPK, particularly related to CREB and mTOR activity is important in cell survival. This study examined the effects of Glu on transcript levels of two CREB target genes, Bcl2 and neuritin [58]. Neuritin is required for neurite outgrowth [59], [60], [61]. Bcl2 is well-studied as an anti-apoptotic factor [62], [63], [64], [65] that can be neuroprotective [66], [67], [68], [69]. Thus, it is possible that strong upregulation of Bcl2 may confer protection on the SCN2.2 cells. However, in our study, we did not find any change in the neuritin mRNA levels with Glu treatment in either the GT1-7 or SCN2.2 cells (except at 36 h, Fig. 5A). Neuritin protein levels also remained unchanged in the SCN2.2 and GT1-7 cells with Glu treatment (Fig. 5A). As opposed to our expectation, Bcl2 mRNA was initially increased in the GT1-7 cells, but showed no change with time in the SCN2.2 cells (Fig. 5B). We speculate that the temporary increase in Bcl2 mRNA in GT1-7 cells may represent an initial protective response, which is overwhelmed by continued Glu toxicity. Surprisingly, while the Bcl2 mRNA levels remained unchanged with Glu treatment in the SCN2.2 cells, Bcl2 protein was significantly increased in the SCN2.2 cells at 48 h. This suggests an additional mechanism activated downstream of ERK/MAPK signaling, which provides neuroprotection to the SCN2.2 cells against Glu.

Bid is a pro-apoptotic molecule whose cleavage triggers formation of the Bax channel [70], thus allowing cytochrome-c to be released from the mitochondria into the cytosol [71]. This in turn results in formation of the apoptosome, leading to caspase 3 activation and ultimately, cell demise. We found that Glu treatment resulted in a strong increase in the Bid mRNA levels in the GT1-7, but not in the SCN2.2 cells (Figure 5C). Bid mRNA levels were significantly increased over a period of 12 to 48 h in the Glu-treated GT1-7 cells but remained steady in the SCN2.2 cells and did not change with respect to untreated control (Figure 5C). The cleaved fragment (activated) Bid appeared in the Glu-treated GT1-7 cells at 48 h, but not in the untreated control (Figure 5C). We were unable to find an appropriate antibody for determining cleaved Bid in SCN2.2 cells. However, these results clearly demonstrate the activation of different pathways in response to Glu in the 2 cell types: a pro-survival ERK/MAPK in the SCN2.2 neurons as opposed to a pro-apoptotic pathway in the GT1-7 neurons.

Porterfield et al., 2007 [72], identified a total of 13 transcripts that were significantly increased in the SCN 30 min after administration of a light pulse. Among these, of interest are genes such as Pim3 (a Ser/Thr kinase), Btg2 and Klf4, all of which have established anti-apoptotic functions in non-neuronal systems. Their role in neurons, more specifically in the SCN neurons, is currently unknown. Future studies should attempt to explore the significance of these genes in SCN neurons.

Functional NMDARs on the post-synaptic SCN neurons [73], [74] are required for the physiological response to nocturnal light in the SCN [75], [76]. NMDAR inhibition in the SCN blocks the effects of light in vivo and its correlate, Glu, in vitro [75], [76]. However, innumerable studies have implicated the NMDARs in mediating Glu excitotoxicity in various neuronal populations [77], [78]. In the present study, NMDA and Glu induced cell death in the GT1-7 neurons, while leaving the SCN2.2 neurons unaffected. Glu and NMDA toxicity in GT1-7 cells was blocked by the NMDAR antagonist MK-801. Furthermore, SCN2.2 cell death induced in the presence of NMDA and the ERK/MAPK inhibitor was blocked by MK-801. These data suggest that NMDAR signaling lies upstream of the ERK/MAPK signaling response to Glu in the SCN2.2 cells.

The complex nature of cell signaling pathways suggests the possibility that numerous signaling events may be involved in conferring resistance to excitotoxicity in SCN2.2 cells. SCN neurons project onto largely GABAergic, neurons within and outside the nucleus [79]. This raises the possibility that the SCN neurons *in vivo* are able to tolerate large glutamate release simply because this excitatory transmission serves largely to increase inhibition. However, in the current study, we have used SCN2.2 cells which lack the enzyme needed to synthesize GABA [80]. Thus, it is unlikely that release of GABA explains all of the endogenous resistance to excitotoxicity in the SCN2.2 cells. Future studies will be performed in vivo to explore the role of GABA neurotransmission in determining resistance of SCN neurons to excitotoxic damage.

Another alternative is that SCN2.2 cells simply lack NMDARs and are incapable of responding to an excitotoxic insult. Experiments performed by Menger et al in 2005, indentified transcripts associated with glutamatergic signaling [80]. Previous work from our lab has established the presence of transcripts (NR1, NR2A and NR2B) and protein products (NR1 and NR2A/NR2B) of NMDAR subunits in the SCN2.2 cells [1]. In the same study, we used a luciferase reporter assay to demonstrate the functional activity of the NMDARs in SCN2.2 cells. The luciferase reporter was linked to DNA regions known to be activated in response to glutamate in the SCN. Glutamate treatment increased the luciferase activity in the SCN2.2 cells; this was attenuated by the NMDAR antagonists MK-801 and AP-5. Collectively, these results serve to establish that the SCN2.2 cells are capable of responding robustly to glutamate.

It is common knowledge that the SCN in vivo is enriched in astrocytes. This raises the possibility that rapid glutamate uptake by glia prevents SCN neurons from exposure to excitotoxic damage. However, it should be noted that in the current study, we have used the SCN2.2 cell line, which is typically comprised of about 95% neurons and 5% glia. While culturing SCN2.2 cells, we use cell culture flasks which have surfaces coated with Poly D-Lysine. This prevents rapid glial proliferation in vitro. Also, a recent study from our lab [1] demonstrated that Inhibition of glutamate transporters did not increase the susceptibility of SCN2.2 cells to glutamate excitotoxicity. It is therefore unlikely that glial uptake of glutamate is the sole mechanism for protection of SCN2.2 cells from excitotoxicity.

SCN and their immortalized counterparts, SCN2.2 cells, are commonly investigated for their rhythmic nature. Whether rhythmicity contributes to protection from excitotoxicity remains an open question. It is possible ERK, p38 and SAPK/JNK have different peaks of activation and that the timing of their activation is key to determining the resistance to excitotoxic damage. The present study did not attempt to address this question. In our hands, cultures of SCN2.2 cells are arrhythmic unless a stimulus such as serum shock is first used to synchronize the culture. All experiments for this manuscript were performed on unsynchronized cultures. It would indeed be interesting to determine whether the timing of activation of the MAPK proteins render the SCN2.2 cells more or less susceptible to excitotoxic damage, and these experiments could easily be performed in the future in synchronized SCN2.2 cells.

Several lines of evidence support the similarity and disparity of the SCN2.2 cells with the SCN in vivo. SCN lesions result in loss of circadian rhythms, which can be restored by implantation of SCN2.2, cells in vivo [81]. A study performed by Menger GJ, et al in 2005 [80] performed exhaustive profiling of genes in the SCN2.2 cells and compared them to the SCN in vivo. They found a significant overlap between the expression patterns of numerous genes between these two cell types. When they synchronized the SCN2.2 cells, they found that many of the same genes that were rhythmic in rat SCN were also rhythmic in the SCN2.2 cells. It would be interesting to determine which of these genes are also responsive to light-induced transcription and have a role in neuroprotection.

**Figure 6:**
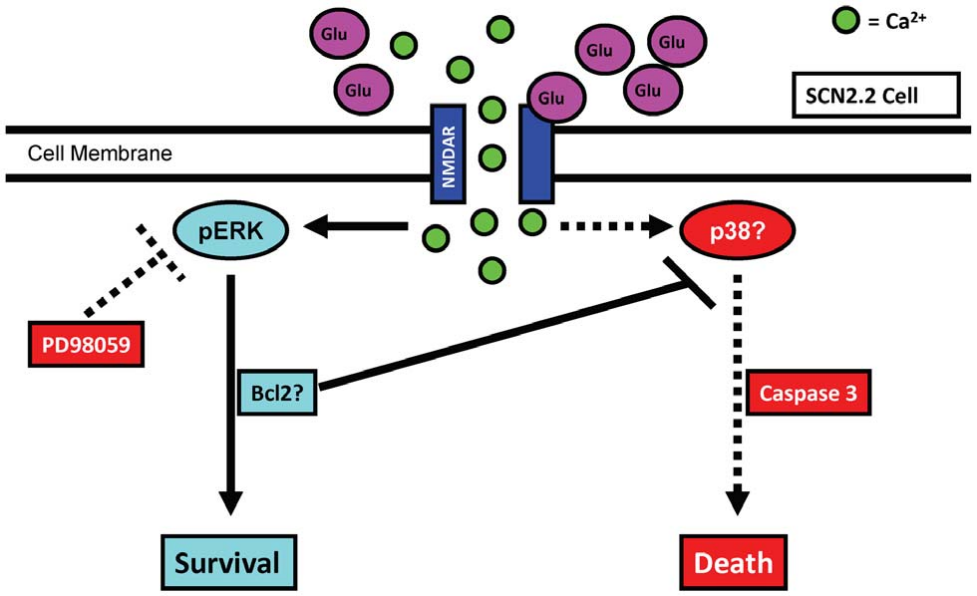
Hypothetical Model for Neuroprotection in SCN2.2 Cells. Glu activates NMDAR, causing an increase in calcium into the cell. Under normal conditions, this leads to a phosphorylation and activation of ERK/MAPK and ultimately cell survival. Bcl2 protein levels are increased, partially explaining the failure to activate caspase 3 and undergo apoptosis in response to excitotoxin. When ERK/MAPK is inhibited with PD98059, this survival pathway is blocked. Caspase 3 activity rises, and the cells undergo apoptosis. The cell signaling pathway connecting NMDAR activity and cell death under conditions of ERK/MAPK inhibition is unknown at present, but it is possible that activation of p38/MAPK as well as reduction in Bcl2 lead to caspase activity and cell death.

Our data, in the context of the current literature, allow construction of a hypothetical model for neuroprotection in the SCN (Figure 6). Activation of NMDARs on SCN neurons results in robust and prolonged activation of ERK/MAPK, which, in turn, promotes activation of downstream anti-apoptotic factors (such as Bcl2) and prevents SCN2.2 cell death. Blocking ERK/MAPK enables Glu to induce excitotoxicity by activating caspase 3-dependent apoptosis. Further experimentation into these mechanisms will help to determine the differences between signaling pathways activated in cells resistant to Glu toxicity as opposed to cells that are susceptible to Glu insult. Thus, future studies will build upon the current findings in moving toward the development of novel therapeutic strategies in the battle against neurodegeneration.

## Materials and Methods

### Chemicals

Glu (an excitotoxin), PD98059 (ERK/MAPK inhibitor), MK-801 (use-dependent NMDAR antagonist) and NMDA (an excitotoxin) were purchased from Sigma.

### Cell culture

The SCN2.2 cells (a gift from Dr. D. Earnest, Texas A&M University) were cultured in Minimum Essential Medium (MEM), supplemented with 7.5% Bovine growth serum (BGS), 10,000 Units/ml penicillin, 10,000 Units/ml streptomycin, 250 µg/ml fungizone on poly-D-lysine-coated T75 flasks. The GT1-7 cells (a gift from Dr. P. Mellon, University of California, San Diego) were cultured in Dulbecco's Modified Eagle Medium (DMEM) supplemented with 7.5% BGS, 4.5 gm/L glucose, 2 mM L-glutamine and 10,000 Units/ml penicillin, 10,000 Units/ml streptomycin, 250 µg/ml fungizone, on uncoated plates. Cells were incubated at 37°C with 5% CO_2_. All cultures were grown to 50% confluence before treatment.

### Live vs. dead Total cell analysis

Cells were plated in 96-well plates at 5000 cells per well. Treatments were initiated when cultures reached 50% confluence. Inhibitors were added 1 h prior to onset of treatment and maintained throughout the treatment period. Fresh medium was placed in the control (untreated) wells. The Live/Dead assay kit (Invitrogen) was used to analyze cell viability. In this assay, the cell membrane permeable dye Calcein AM is metabolized to the fluorescent green dye Calcein which is cell non-permeable. Dead cells are measured by the DNA binding dye Ethidium homodimer-1, which fluoresces red upon binding to DNA. This dye cannot enter the cell unless the membrane is already damaged, and so does not tag healthy cells. Optical density was measured for each sample using the Fluoroskan 96 well plate reader (MTX Lab Systems). For the green Calcein staining living cells, excitation is 485 nm and emission is 538 nm. For the red Ethidium homodimer-1 staining dead cells, excitation is 544 nm and emission is 590 nm.

### MTS Assay

Cells were plated in 96 well plates at 5000 cells per well and treated at 50% confluence. Inhibitors were added 1 h before treatment was initiated and maintained throughout the treatment period. Fresh medium was placed in the control (untreated) wells. For the assay, 100 µl of medium was removed from each well. 20 µl of MTS reagent (CellTiter 96 Aqueous One Solution Cell Proliferation Assay, Promega) was added to each well. The cells were incubated at 37°C in 5% CO2 for 1–4 h, in accordance with manufacturer's protocol. After color development, the plate was read at 490 nm (800EL Universal Microplate Reader, Bio-Tek Instruments, Inc). Optical densities were used to determine % metabolic activity with respect to untreated control.

### TUNEL assay

Cells were grown in T25 flasks and treated at 50% confluence. Inhibitors were added 1 h before treatment was initiated and maintained throughout the treatment period. Fresh medium was placed in the control (untreated) flasks. The TUNEL assay was performed with the FragEL DNA Fragmentation Detection Kit (Calbiochem), at the end of 48 h of Glu treatment, according to the manufacturer's specifications. Coverslips were mounted with DAPI in antifade solution. Negative controls included omission of the TdT enzyme. Alternatively, TUNEL was performed with the Apoptag Apoptosis Detection System (Chemicon, Temecula, CA) according to the manufacturer's specifications. Slides were mounted with DAPI in antifade solution. Negative controls included omission of the TdT enzyme or the anti-Digoxigenin antibody. Images were taken using the Zeiss Axiovert 200 microscope equipped with a digital camera. The entire set of treatments followed by the TUNEL assay was performed in triplicate. Cells positive for fluorescein stained the apoptotic TUNEL-positive cells. Total cells were counted by two independent observers, each blind to experimental condition. Apoptotic cells were quantified using the formula:
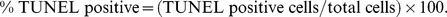



### Protein extraction for Western blotting

Medium was removed and cells were washed with PBS. Tissue Protein Extraction Reagent (T-PER; Pierce, Rockford, IL), containing 1× Protease Inhibitor (Roche, Indianapolis, IN), was added. Phosphatase inhibitors (Phosphatase inhibitor cocktail 1, Sigma and Phosphatase inhibitor cocktail 2, Sigma) were also added to the extraction reagent (1 µl of each for every 100 µl of the extraction reagent). Cells were scraped into the extraction solution and placed into microfuge tubes. Tubes were spun at 10,000 rpm for 5 min (at 4°C) to precipitate cellular debris. Supernatants were placed in fresh microfuge tubes and stored at −80°C. Protein concentration in supernatants was determined by using the Pierce BCA Protein Assay Kit (Thermo Scientific) as per the manufacturer's protocol.

### Western Blotting

Cells were grown in T25 flasks and treated at 50% confluence. Inhibitors were added 1 h prior to treatment and were present throughout the treatment period. Fresh medium was placed in the control (untreated) flasks. At the end of the treatment period, the medium was removed and the protein was extracted and assayed as described above. 80–100 µg of protein was resolved by SDS-PAGE and transferred to a nitrocellulose membrane. The membrane was blocked with 5% non-fat dry milk (LabScientific, Inc.) dissolved in 0.05% Tris buffered saline – Triton (TBS-T; 50 mM Tris-HCl, 150 mM NaCl, pH 7.5+50 ml/100 ml Triton-X 100) at room temperature for 1 h. Membranes were washed with 0.05% TBS-T and incubated overnight at 4°C in primary antibody: phospho-p44/42 MAPK (Erk1/2) (Thr202/Tyr204) rabbit monoclonal antibody (1∶2000) (Cell Signaling); phospho-p38 MAPK mouse antibody (1∶2000) (Cell Signaling); p44/42 MAPK (Erk1/2) mouse monoclonal antibody (1∶2000) (Cell Signaling); Beta-Actin mouse antibody (1∶5000) (Sigma) or tubulin mouse antibody (1∶5000) (Sigma). The latter two were used as loading controls. The primary antibody solution was removed and the membrane was washed 3 times with 0.05% TBS-T, for 10 min each. The membrane was then treated with anti-rabbit antibody (Odyssey LiCor, IR800) (1∶5000) or anti-mouse antibody (Odyssey LiCor, IR700) (1∶5000) for 1 h at room temperature. The secondary antibody solution was removed and the membranes were washed 3 times with 0.05% TBS-T, for 10 min each. All the antibody dilutions were made in SuperBlock Blocking Buffer in TBS (Thermo Scientific). The blots were analyzed using the LiCor Odyssey infrared imaging and densitometric analysis was performed using the LiCor Odyssey software.

### Caspase 3 activity assay

Cells were grown in T75 flasks. Treatments were initiated at 50% confluence. At the end of 48 h, the medium was removed and the cells were washed with phosphate buffered saline (PBS). Caspase 3 activity assay was performed using the caspase 3 Colorimetric Assay kit (R&D Systems), as per the manufacturer's protocol. A small portion of the lysate from the samples was saved for protein assay. Protein quantification was done using the Micro BCA Protein Assay Kit (Thermo Scientific), as per the manufacturer's protocol. Caspase 3 activity per mg of protein was measured and relative increase or decrease was measured using the untreated control as a standard. A standard curve was prepared. All experiments were performed in triplicate.

### Immunocytochemistry

Cells were cultured on coverslips in a 24-well plate. The treatments were done at 50% confluence. Inhibitors were added 1 h prior to onset of treatment. At the end of the 48 h treatment period, the medium was removed. Cells were washed with 1 ml of Dulbecco's Phosphate Buffered Saline (DPBS) per well. The DPBS was removed and 200 µl of 2% paraformaldehyde (PFA) in DPBS was added to each well. The cells were fixed for 15 min in PFA. The cells were rinsed 3 times, 5 min each, with PBS and blocked for 1 h with 5% goat serum in 0.3% PBS-Triton-X 100 (PBS-T). Cells were then incubated overnight at 4°C, in primary antibody. The cells were washed 3 times, 10 min each, in PBS-T. The secondary antibody treatment was done for 1 h at room temperature in the dark. Cells were again washed with PBS-T, 3 times, 5 min each. The coverslips were then mounted onto slides using Vectashield.

### Treatments for mRNA quantification

Cells were cultured in 6 well plates. At 50% confluence, 10 mM Glu was added to half the wells. Medium without Glu was placed in the untreated control wells. At each time-point (1, 6, 12, 18, 24, 36, 48, 60 and 72 h) the medium was removed. Cells were washed with 0.01 M PBS. 1 ml TRIzol (Invitrogen) solution was added per well. The plate was kept at −80°C until analysis.

### RNA extraction

Samples were incubated in TRIzol at room temperature for 15 min to allow dissociation of nucleoprotein complexes. RNA was extracted into the aqueous phase by addition of 0.2 ml chloroform per 1 ml TRIzol. The chloroform/TRIzol mixture was vortexed for 15 s, kept at room temperature for 2–3 min and centrifuged at 12,000 rpm for 15 min. The colorless aqueous phase that contains RNA was transferred to a fresh RNase-free tube. Isopropyl alcohol (0.5 ml/1 ml TRIzol originally used) was added. The co-precipitate Glycoblue (1 µl) was added to each tube to aid in visualization of the RNA pellet. Linear acrylamide (1 µl) was added to increase RNA yield. This mixture was kept at −20°C overnight. Samples were centrifuged for 10 min at 12,000 rpm. The RNA pellet was washed with 1 ml of 75% ethanol (in DEPC-water) and air dried. The RNA pellet was then dissolved in DEPC-water (Fisher Bioreagents). Concentration of the RNA was determined using the NanoDrop ND-1000 UV spectrophotometer at 260 nm and 280 nm. Purity of the samples was determined by checking the 260/280 and the 260/230 ratios. Negative controls included omission of RNA template and omission of the reverse transcriptase (RT) enzyme.

### Quantitative PCR

The Bid primers from mouse were designed using NCBI Primer Blast; forward primer: GCCGAGTGTGGCTCCGCAAA and reverse primer: CCGGAACCGTTGCTGACCT. All other primers were purchased from Qiagen and the sequences are proprietary. qPCR reactions were performed in duplicate in the Smart Cycler (Cepheid). Standard curves were performed for each of the primers as previously described [1]. Results of the standard curves are given in Table 2.

**Table 2:** qPCR Primers and Standard Curves.

Gene	Species	Source of Primers	Efficiency	R^2^	Equation
Bcl2	Rat	Qiagen QuantiTect	1.167	0.987	Y = −0.897X+33.69
Bid	Rat	Qiagen QuantiTect	1.018	0.992	Y = −0.987X+32.29
Nrn	Rat	Qiagen QuantiTect	1.377	0.956	Y = −0.899X+30.84
Bcl2	Mouse	Qiagen QuantiTect	0.996	0.990	Y = −1.003X+34.62
Bid	Mouse	Invitrogen	1.080	0.989	Y = −0.9462X+32.49
Nrn	Mouse	Qiagen QuantiTect	0.854	0.989	Y = −1.123X+34.75

Primers for genes from rat (for the SCN2.2 cell line) and mouse (for the GT1-7 cell line) are shown. The sequences of all primers from Qiagen are proprietary; the sequences for the mouse Bid primers are given in the Materials and Methods section. Standard curves were prepared from serial dilutions of total RNA from each tissue. Efficiency is calculated as 2^−(1/slope)^. The values for R^2^ and the equation for the line were derived from Excel.

### Statistics

Two-way ANOVA with Bonferroni's post hoc test for multiple comparisons was used to analyze effects of cell type and treatment. Effects of cell treatment on a single cell type were analyzed with repeated measures ANOVA and Tukey's post hoc test. Means of control and 48 h treatment were compared with paired *t* test. All statistics were performed with GraphPad Prism 5.0 software.
